# CircAP2A2 acts as a ceRNA to participate in infantile hemangiomas progression by sponging miR‐382‐5p via regulating the expression of VEGFA

**DOI:** 10.1002/jcla.23258

**Published:** 2020-02-24

**Authors:** Xiaoqi Yuan, Yanan Xu, Zhiqiang Wei, Qi Ding

**Affiliations:** ^1^ The Ningbo Women and Children's Hospital Ningbo China; ^2^ Department of Diagnosis Ningbo Diagnostic Pathology Center Ningbo China

**Keywords:** circAP2A2, circRNA, infantile hemangiomas, miR‐382‐5p, VEGFA

## Abstract

**Background:**

Increasing evidences reveal that circular RNAs (circRNAs) play crucial functions in cancer development. However, the expression pattern and roles of circRNAs in infantile hemangiomas (IH) remain unclear.

**Methods:**

In this study, qRT‐PCR was performed to determine the expression of circAP2A2, miR‐382‐5p, and VEGFA in IH tissues and cell lines. Moreover, MTT assay, colony formation, transwell assay, and Western blot analysis were conducted to assess the function of circAP2A2 or miR‐382‐5p on cell proliferation, and migration in vitro, respectively. Also, dual luciferase assay was used to confirm the interactions among circAP2A2, miR‐382‐5p, and VEGFA.

**Results:**

CircAP2A2 was confirmed to be highly expressed in IH. CircAP2A2 knockdown or miR‐382‐5p overexpression decreased the proliferation, colony formation, migration, and invasion of HemECs and HUVEC cells.

**Conclusion:**

CircAP2A2 could promote proliferation and invasion of IH by regulating miR‐382‐5p/VEGFA axis.

## 1 INTRODUCTION

Infantile hemangiomas (IH) are the most common soft‐tissue tumors of infancy. The prevalence of infantile hemangioma in mature neonates is around 4.5%.^1^ Infantile hemangiomas have been well characterized by its distinctive presentation, namely rapid growth during infancy followed by subsequent regression in early childhood.^2^ This lesion is usually harmless; however, ~10% of lesions (those that compromise vision or vital systems like the airways) require treatment during the proliferative stage. Periocular lesions are of special concern as they pose a threat to the development of normal visual sensory function, leading to amblyopia in 43%‐60% of cases.^3^, ^4^ Approximately 80% of lesions affect the head and neck. However, no available clinical biomarker is used to sensitively and specifically diagnose IH.^5^ Thus, the pathogenesis and possible therapeutic targets of IH should be investigated.

In the past, circular RNAs (circRNAs) are a class of long, non‐coding RNAs molecules. However, recent researchers have discovered that circRNAs can be translated.^6^ circRNAs are characterized by covalently closed continuous loop which have no 5′‐3′ polarity and contain no polyA tail.^7^ Emerging evidences have indicated that thousands of endogenous circRNAs are present in mammalian cells.^8^ One representative function of circRNA is that it could act as a microRNA (miRNA) sponge to regulate the stability and/or translation efficiency of other RNAs, and such a circRNA is called a competing endogenous RNA (ceRNA).^9^ Furthermore, circRNAs might play important roles in colon cancer,^10^ pancreatic ductal adenocarcinoma,^11^ gastric cancer^12^ and breast cancer^13^ and serve as diagnostic or predictive biomarkers of cancer.

Infantile hemangiomas is the result of dysregulation of both vasculogenesis and angiogenesis.^2^ Despite extensive literature, the pathogenesis is still not clear. Recently, there is a most likely hypothesis that can explain the pathogenesis, involving hypoxic stress as the triggering signal,^14^ by inducing overexpression of angiogenic factors (such as VEGF) via the HIFα pathway.^15^ In response to VEGF overexpression, stem cells (expressing CD133), naturally present or recruited in fetal skin, proliferate and differentiate into immature endothelial cells (expressing CD31), but also pericytes (expressing SMA), dendritic cells (expressing factor XIIIa), and mesenchymal cells have the potential adipogenic activities.^16^ Therefore, VEGFA (vascular endothelial growth factor A) plays a key role in pathological angiogenesis and angiogenesis of IH.^17^ Furthermore, some researchers have demonstrated that circRNA could directly bind to miRNA and regulates VEGF expression in bladder carcinoma.^18^ Considering the critical role of circRNAs and VEGF in cancer, it has been proposed that whether circRNAs could directly bind to miRNA by regulation of VEGFA expression in IH. Based on the previous microarray assay of circRNA in infantile hemangiomas,^19^ we screened circAP2A2 and verified its high expression in IH by qRT‐PCR. By using the bioinformatics software, miR‐382‐5p was predicted to be a target of circAP2A2, and VEGFA was suggested to be a putative target gene of miR‐382‐5p. Therefore, we speculated that the circAP2A2/miR‐382‐5p/VEGFA regulatory feedback circuit could affect the proliferation, migration, and invasion of IH cells. By exploring the hypothesis, we may gain insights into the pathogenesis of IH and provide new therapeutic targets for the treatment of IH.

## 2 MATERIALS AND METHODS

### 2.1 Human tissue specimens

Infantile hemangiomas samples were collected at the Ningbo Women and Children's Hospital from 2015 to 2019. The excised samples were immersed overnight in RNAstore (CWBIO) at 4°C and preserved at −80°C.

### 2.2 Cell culture

Human hemangioma‑derived endothelial cells (HemECs) and human umbilical vein endothelial cells (HUVEC) were cultured in RPMI 1640 medium (Hycolon) supplemented with 10% (v/v) fetal bovine serum and 100 U/mL penicillin‐streptomycin (Sigma‐Aldrich).

### 2.3 RNA extraction and reverse transcription

Total RNAs were extracted from tissues or cultured cells using TRIzol reagent (Invitrogen), according to the manufacturer's instructions. The extracted RNAs were reverse transcribed into cDNAs using ReverTra Ace qPCR RT Kit (TOYOBO).

### 2.4 Treatment with RNase R and RNA localization experiment

RNase R treatment was carried out for 15 minutes at 37°C using RNase R (Epicenter) 3 U/mg. For RT‐PCR, the treated RNA was directly reverse transcribed into cDNA. Cytoplasmic and nuclear RNA isolations were performed using a PARIS Kit (Invitrogen), following the manufacturer's instructions. The extracted RNAs were reverse transcribed into cDNAs.

### 2.5 Quantitative real‐time PCR

qRT‐PCR was performed using the Light Cycler 480 SYBR Green I Master (Roche). The PCR conditions were 95°C for 5 minutes, 95°C for 10 seconds, 56°C for 20 seconds, and 72°C for 30 seconds with the latter three steps repeated for 45 cycles. The primer sequences of circAP2A2 were 5′‐CCTGGCTCCGAAGACAACTT‐3′ (forward primer) and 5′‐GGCGTAGACTAAATTCTGCCG‐3′ (reverse primer). β‐actin was 5′‐CACCAGCATCTTTTCCAACC‐3′ (forward primer) and 5′‐AAGGCCGACTCTCCTACACA‐3′ (reverse primer). The relative expression was shown as ∆*C*_t_ value by subtracting the β‐actin *C_t_* value from the circRNA *C*_t_ value. Higher gene expression was indicated by a smaller ∆*C*_t_ value.

### 2.6 siRNAs, miRNA mimic, and transfection experiments

The circAP2A2 siRNAs and the mimic of miR‐382‐5p, as well as the non‐targeting negative control, were purchased from GenePharma. Lipofectamine 2000 (Invitrogen) was used for transfection. Two circAP2A2 siRNAs with sequences of si‐circAP2A2‐1, 5′‐AGAATTTAGTCTACGCCTTCT‐3′ and si‐circAP2A2‐2:5′‐ GCAGAATTTAGTCTACGCCT‐3′, were used for the knockdown experiment.

### 2.7 MTT Assay

Cell proliferation was determined by Thiazolyl Blue (MCE) assay. In brief, 20 μL MTT (5 mg/mL in PBS) was added to the medium and incubated at 37°C for 4 hours. The supernatants were carefully aspirated, and 100 μL DMSO was added to each well. Absorbance values at 490 nm were measured.

### 2.8 Colony formation assay

For the cell colony formation assay, stably transfected cells (1000 per well) were plated in six‐well plates. After culturing for 7‐9 days, cells were fixed with 70% ethanol and stained with 0.5% crystal violet solution. Colonies with more than 50 cells per colony were counted. All experiments were conducted three times in triplicate.

### 2.9 Transwell migration and matrigel invasion assays

The migration and matrigel invasion assays were conducted by using transwell chamber (for migration assay) or transwell pre‐coated matrigel chamber (for invasion assay) according to the manufacturer's protocol (Corning). The homogeneous single cell suspensions (4 × 10^4^ cells/well for migration, 1 × 10^5^/well for invasion) were added to the upper chambers and incubated for 24 hours. The migration and invasion rates were quantified by counting the migration and invaded cells at least three random fields.

### 2.10 Dual luciferase assay

Dual luciferase assay was conducted according to the protocol from the manufacturer (Promega). WT and mutant circAP2A2 and VEGFA were amplified and cloned into the luciferase reporter vector pGL3 (Promega). 293T cells were co‐transfected with luciferase plasmids and miR‐382‐5p mimics. After 48 hours, the activities of firefly luciferase were normalized to that of Renilla luciferase.

### 2.11 Western blot analysis

Total protein was separated by SDS‐polyacrylamide gel electrophoresis (SDS‐PAGE). The proteins in SDS‐PAGE were transferred onto nitrocellulose membranes (GE Healthcare). The membrane was incubated with rabbit anti‐VEGFA (ab46154, 1:1000 dilution; Abcam) primary antibodies overnight at 4°C and then with secondary antibody at room temperature for 1 hour. Proteins of interest were visualized using ECL Plus Western blotting Detection Reagents (Millipore).

### 2.12 Statistical analysis

Unless otherwise stated, all data are shown as mean ± standard error of the mean (SEM). Statistical analysis was performed with two‐tailed *t* tests to compare mean values (Prism; GraphPad Software). A *P*‐value less than .05 was considered statistically significant. *P < .05, **P < .01, ***P < .001.

## 3 RESULT

### 3.1 Screening of candidate circRNAs

**Figure 1:**
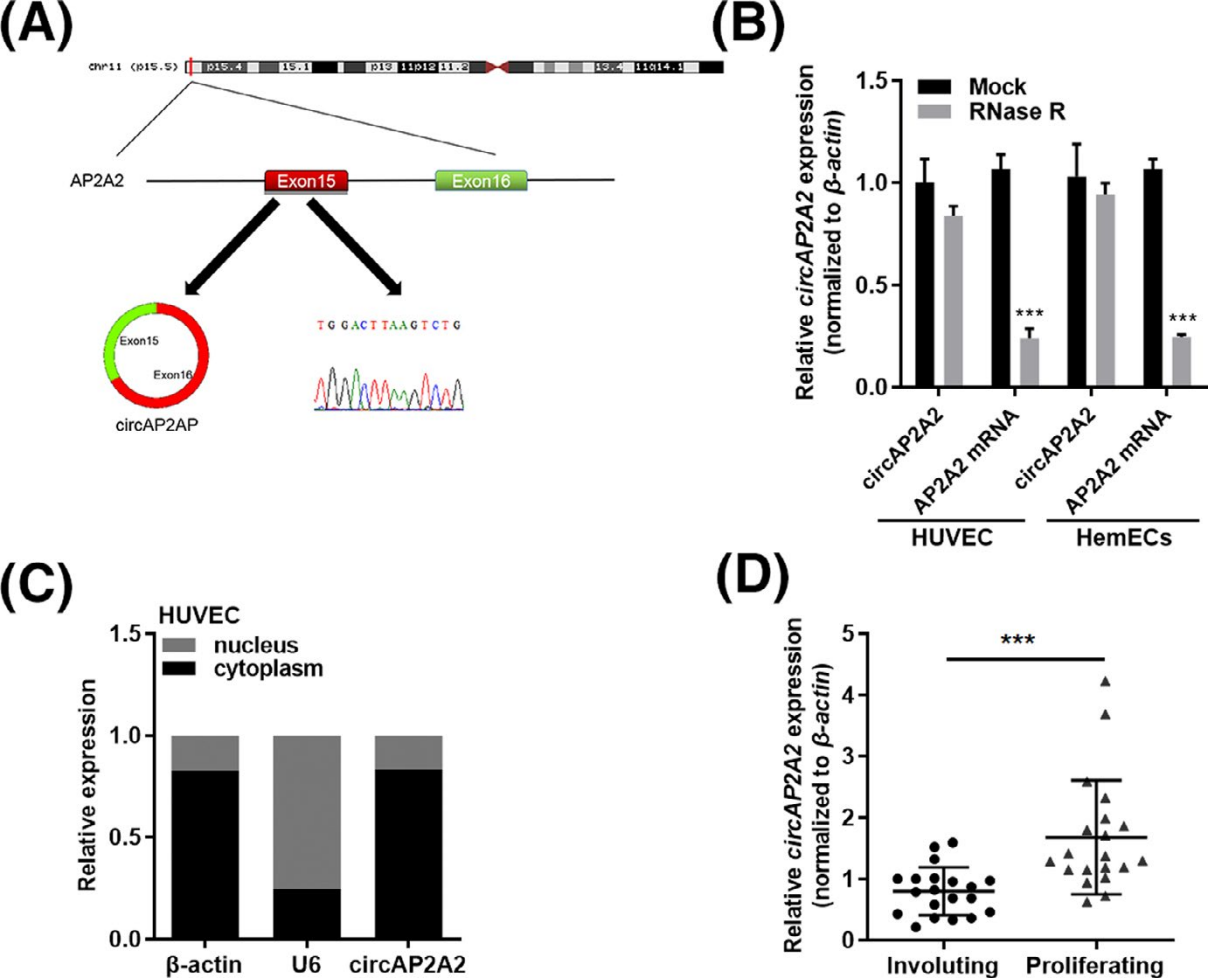
Characterization and expression of circAP2A2 in IH. A, Sequence of circAP2A2 in circBase (upper part) is consistent with Sanger sequencing. B, Circular RNA circAP2A2 is resistant to RNase R treatment in HUVEC cell lines. C, Expression of circAP2A2 in nucleus and cytoplasm by qRT‐PCR. D, Expression of circAP2A2 in paired IH tissues (n = 20)

We selected two aberrantly expressed circAP2A2 and circFAM53P as circRNAs.^19^ In the hemangiomas specimens, circAP2A2 is expressed in hemangiomas by qRT‐PCR, which is consistent with the microarray analysis. Also, circAP2A2 consists of two exons, derived from exons 15 and 16 of AP2A2 gen, with a length of 250 nt (Figure 1A).

### 3.2 CircAP2A2 is highly expressed in hemangiomas and is mainly localized in cytoplasm

We designed a reverse primer spanning the cyclization site and confirmed the single and reverse splicing target fragment of the amplified product by qRT‐PCR and Sanger sequencing based on the circAP2A2 sequence (Figure 1A).

In the second step, we used RNase R digestion to confirm the presence of circAP2A2, and circAP2A2 can tolerate RNase R digestion because RNase R can digest linear RNA and it has no polyA tail. The results of qRT‐PCR showed that there was no significant change in the expression level of circAP2A2 before or after RNase R treatment in HUVEC and HemECs cell lines, but the expression of AP2A2 mRNA was significantly decreased after treatment with RNase R (*P* < .001) (Figure 1B). Then, we used qRT‐PCR to detect the expression of circAP2A2 in normal tissues of 20 pairs of hemangiomas. The expression level of circAP2A2 was more highly in hemangiomas compared with normal tissues (*P* < .05, Figure 1C). Therefore, we confirmed the presence of circAP2A2 in hemangiomas and cell lines. Moreover, it has been found that circAP2A2 was mainly localized in the cytoplasm of HemECs and HUVEC cells by nuclear separation experiments, and by using both U6 (localized in the nucleus) and β‐actin as the control (localized in the cytoplasm) (Figure 1D).

### 3.3 Knocking down circAP2A2 can significantly inhibit the growth, proliferation, invasion, and migration of Hemangioma cells

**Figure 2:**
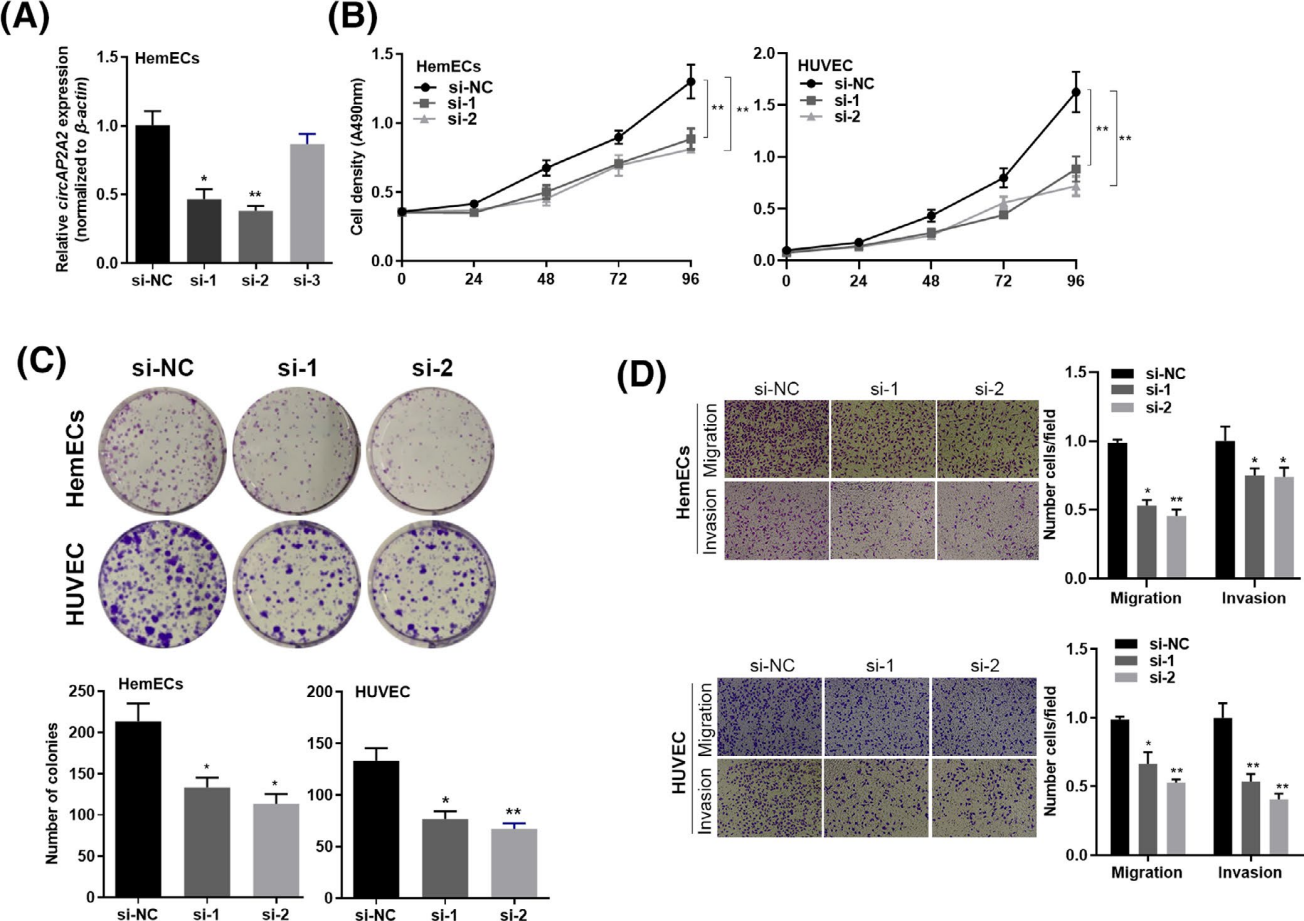
Cytobiology change after treating cells with circAP2A2 siRNA. A, HemECs and HUVEC cell lines are transfected with si‐NC or si‐circAP2A2, and qRT‐PCR analysis demonstrates that the transfection is successful. B, MTT assay is performed to evaluate cell proliferation. C, Colony formation assays are conducted in different treated cells. D, Transwell assay is performed in different treated cells as described in the migration and invasion of HemECs and HUVEC cells. All experiments were conducted three times in triplicate

Previously, we have demonstrated that circAP2A2 is highly expressed in hemangiomas, suggesting that it may have potential carcinogenic functions. Therefore, we transfected circAP2A2 siRNA into HUVEC and HemECs. qRT‐PCR results showed that the expression of circAP2A2 was significantly decreased in the si‐circAP2A2 group transfected with circAP2A2 siRNA compared with the NC group (*P* < .05 and *P* < .01, Figure 2A). The effect of circAP2A2 on cell proliferation was examined by MTT assay, which showed that the cell proliferative ability of si‐circAP2A2 group was significantly decreased compared with NC group (*P* < .01, Figure 2B), indicating that si‐circAP2A2 can inhibit the proliferation of HUVEC and HemECs. Also, the effect of circAP2A2 on cell growth was examined by colony formation assay. The results showed that the cell growth ability of the si‐circAP2A2 group was significantly decreased compared with the NC group (*P* < .05 and *P* < .01, Figure 2C), indicating that si‐circAP2A2 can inhibit HUVEC and growth of HemECs cells. Moreover, the Transwell assay was used to detect cell migration and invasion. The results showed that the ability of cell migration and invasion was inhibited in the si‐circAP2A2 group compared with the NC group (*P* < .05 and *P* < .01, Figure 2D), indicating that si‐circAP2A2 can inhibit migration and invasion of HUVEC and HemECs cells.

### 3.4 CircAP2A2 regulates the expression of miR‐382‐5p

**Figure 3:**
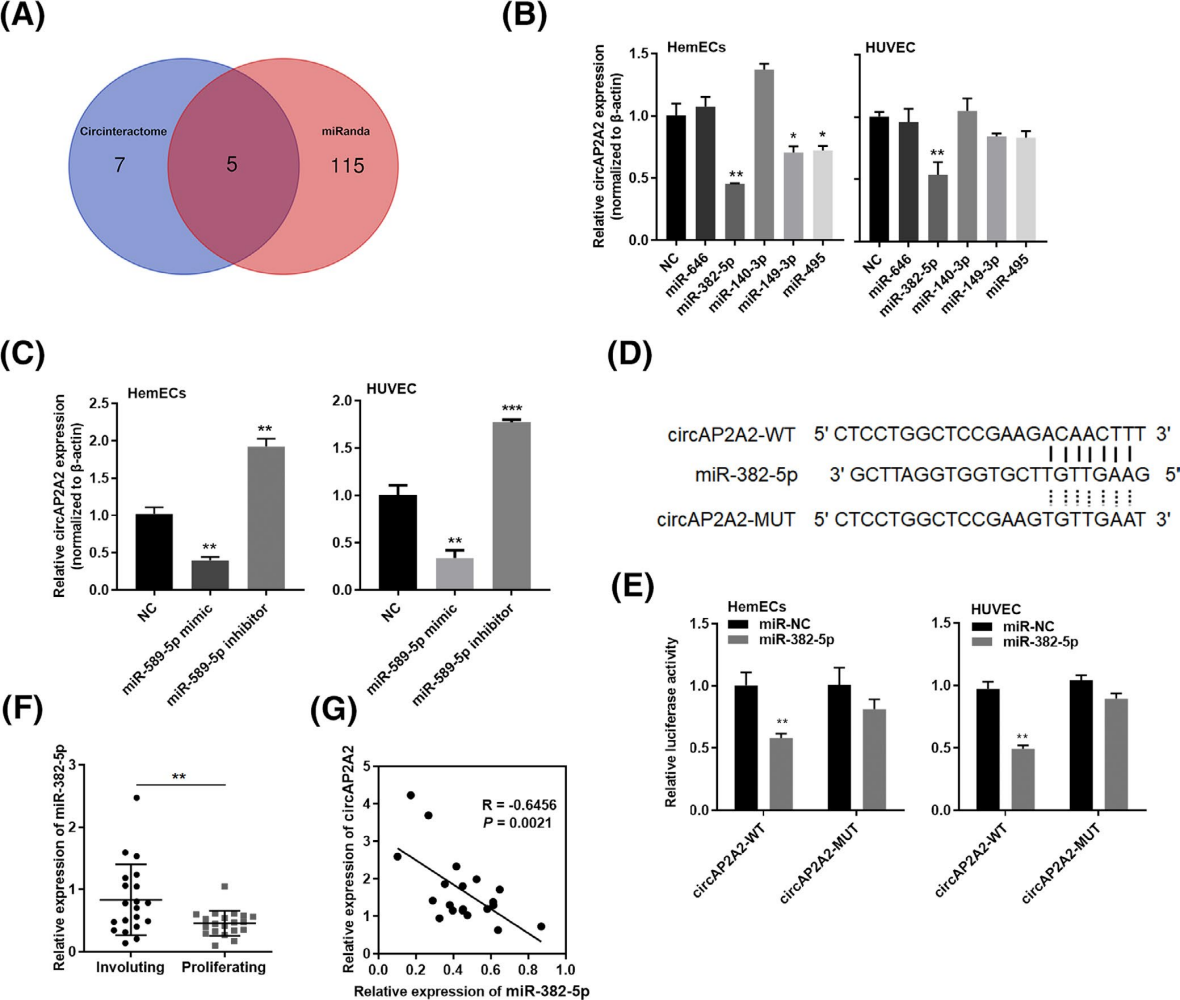
CircAP2A2 serves as a sponge for miR‐382‐5p. A, Putative miRNA binding sites in the circAP2A2 sequence. B, The expression of circAP2A2 after transfection with putative miRNAs through qRT‐PCR. C, The relative expression of circAP2A2 after upregulation or downregulation of miR‐382‐5p. D, Putative miRNA recognition sites are cloned downstream of the luciferase gene. Bottom: mutations in the circAP2A2 and miR‐382‐5p sequences to create the mutant luciferase reporter constructs. E, Luciferase reporter in HemECs and HUVEC cells. Luciferase activity is determined by using dual luciferase assay and is shown as the relative luciferase activity normalized to renilla activity. F, Relative expression of miR‐382‐5p in paired IH tissues. G, Bivariate correlation analysis of the relationship between circAP2A2 and miR‐382‐5p expression level, a significant negative correlation is observed between the expression levels of circAP2A2 and miR‐382‐5p in the same paired samples

We predict the miRNAs of circAP2A2 with possible binding sites with circinteractome and miRanda by crossing two biological softwares (Figure 3A), and a total of five miRNAs were found, including miR‐646, miR‐382‐5p, miR‐140‐3p, miR‐149‐3p, and miR‐495. Detection of circAP2A2 expression after transfection of miRNA mimics, respectively, it was found that only miR‐382‐5p was significantly affected by the negative regulation of circAP2A2 by other miRNAs (Figure 3B). To further investigate whether circAP2A2 has a regulatory effect on miR‐382‐5p, we first upregulated miR‐382‐5p in HemECs and HUVEC cells to reduce circAP2A2 expression. Conversely, downregulation of miR‐382‐5p has increased circAP2A2 expression. These results suggest that circAP2A2 may regulate the expression of miR‐382‐5p by "miRNA sponge action" (Figure 3C).

### 3.5 CircAP2A2 is a "molecular sponge" of miR‐382‐5p to play a regulatory role

We used software to search for the potential binding site of miR‐382‐5p on the circAP2A2 base sequence, and also constructed a wild‐type luciferase vector (PGL3‐circAP2A2‐WT) and a mutant luciferase vector (PGL3‐circAP2A2‐MUT) containing a partial sequence of circAP2A2 (Figure 3D). Next, we transfected miR‐382‐5p mimics and circAP2A2 wild‐type luciferase vectors simultaneously into HUVEC and HemECs cells, respectively. It has been found that the relative activity of dual luciferase was significantly downregulated, while the relative activity of the dual luciferase was not changed, followed by miR‐382‐5p mimics and circAP2A2 mutant luciferase vectors transfection. These experimental results indicate that circAP2A2 binds to circAP2A2, and circAP2A2 regulates the expression of miR‐382‐5p by "miRNA sponge action"(Figure 3E). Moreover, the correlation analysis of the specimens was performed. We detected the expression of miR‐382‐5p by qRT‐PCR in 20 (Figure 3F) pairs of fresh hemangiomas and corresponding adjacent tissues (Figure 3G), and found that there were significantly low expression levels of miR‐382‐5p in hemangiomas. Statistical analysis showed that miR‐382‐5p is negatively correlated to circAP2A2 in this 20 pairs of tissues (Figure 3G).

### 3.6 VEGFA is a direct target of miR‐382‐5p

**Figure 4:**
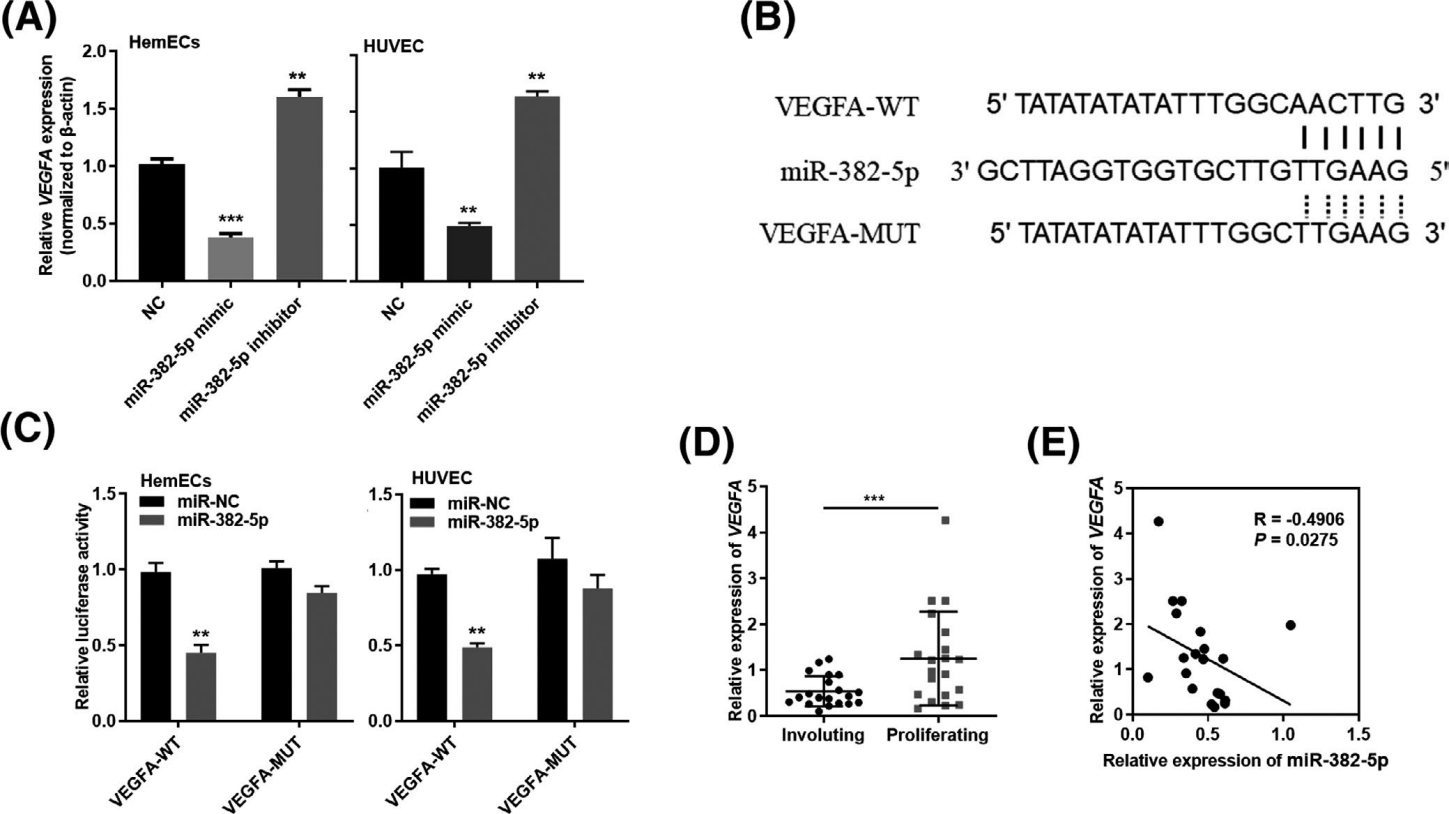
MiR‐382‐5p is targeted to VEGFA. A, HemEC and HUVEC cell lines are treated with miR‐NC or miR‐382‐5p, and qRT‐PCR is used to detect the expression of VEGFA. B, The flowchart of dual luciferase reporter constructs of PGL3‐VEGFA ‐WT/MUT. C, Luciferase activity is determined by using dual luciferase assay after treated with miR‐382‐5p mimics and PGL3‐VRGFA‐WT/MUT. D, VEGFA expression in mRNA level is measured by qRT‐PCR in paired IH tissues. E, Bivariate correlation analysis of the relationship between miR‐382‐5p and VEGFA expression level, a significant negative correlation is observed between the expression levels of miR‐382‐5p and VEGFA in the same paired samples

We found that miR‐382‐5p has potential interaction with VEGFA through a biological website and literature search. Therefore, we explored whether miR‐382‐5p can play a role in the regulation of VEGFA expression in hemangiomas cells. Upregulation of miR‐382‐5p in HemECs and HUVEC cells reduced VEGFA expression. Conversely, downregulation of miR‐382‐5p increased VEGFA expression (Figure 4A). To further explore whether miR‐382‐5p can directly target the 3' UTR region of VEGFA in hemangiomas cells, We constructed a wild‐type luciferase reporter plasmid (PGL3‐VEGFA‐WT) containing two potential binding sites for the VEGFA 3'UTR region targeting sequence and a luciferase reporter plasmid (PGL3‐VEGFA‐ MUT) (Figure 4B). By simultaneously transfecting miR‐382‐5p mimics and VEGFA wild‐type luciferase vectors into HUVEC and HemECs cells, it was found that the relative activity of the dual luciferase was significantly downregulated, while the relative activity of the luciferase vector and the dual luciferase did not change, followed by miR‐382‐5p mimics and VEGFA mutants transfection. (Figure 4C). These experimental results demonstrate that VEGFA can bind to circAP2A2. In the meanwhile, we detected the expression of VEGFA by qRT‐PCR in 20 pairs of fresh hemangiomas and corresponding paracancerous tissues (Figure 4D), and found that it was significantly upregulated in hemangiomas. Statistical analysis showed that miR‐382‐5p was negatively correlated with VEGFA in these 20 pairs of tissues (Figure 4F).

### 3.7 CircAP2A2/miR‐382‐5p/VEGFA axis regulates proliferation, invasion, and metastasis of hemangiomas cells

**Figure 5:**
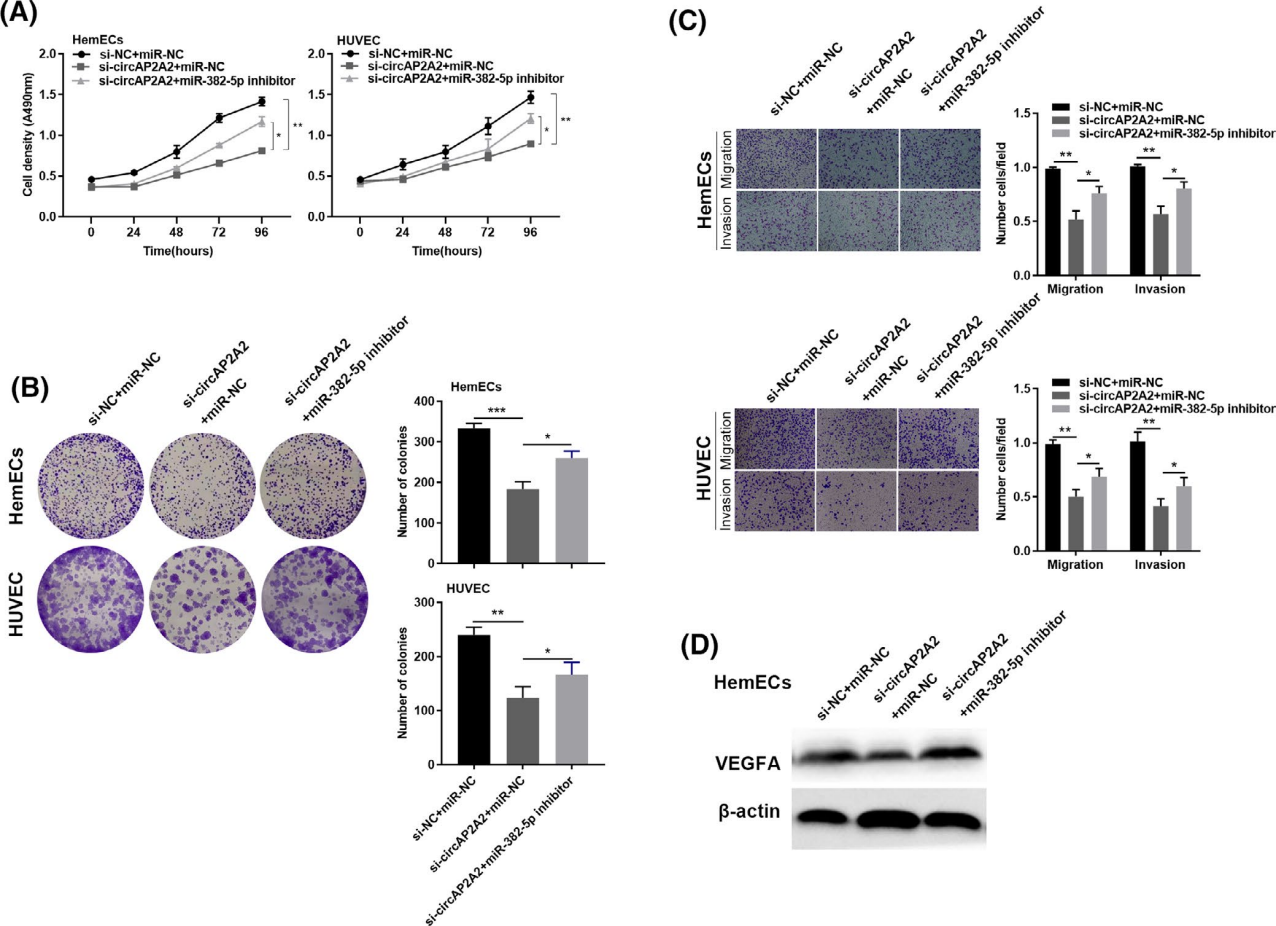
CircAP2A2 suppresses cells progression by sponging miR‐382‐5p and upregulating VEGFA expression. A, HemECs and HUVEC cell lines are co‐transfected with si‐circAP2A2 and miR‐382‐5p inhibitor, proliferation ratio of HemECs and HUVECX cell lines is measured by MTT. B, Colony formation assays are conducted in different treated cells. C, Migration and invasion were detected by transwell assay. D, VEGFA protein expression level is analyzed through Western blot. All experiments were conducted three times in triplicate

We used MTT assay to detect cell growth, showing that transfection of miR‐382‐5p inhibitor promotes cell growth, but inhibition of miR‐382‐5p and downregulation of circAP2A2 can restore cell growth (Figure 5A). The results of cell clone formation experiments showed that transfection of miR‐382‐5p inhibitor inhibited the number of cloned cells, but knockdown of miR‐382‐5p while downregulating circAP2A2 restored the number of cloned cells (Figure 5B). Transwell experiments showed that downregulation of miR‐382‐5p promoted cell migration and invasion, and downregulation of miR‐382‐5p while downregulating circAP2A2 restored cell migration and invasion (Figure 5C). Western Blot experiments also showed that a decrease in VEGFA expression caused by knockdown of circAP2A2 was partially restored by knocking down miR‐382‐5p (Figure 5D). These results indicate that circAP2A2 regulates the proliferation, invasion, and metastasis of hemangiomas cells through the circAP2A2/miR‐382‐5p/VEGFA pathway.

## 4 DISCUSSION

CricRNAs are a new class of RNAs molecules that are highly conserved in evolution and structurally stable and commonly exist in various species.^20^, ^21^ Moreover, cricRNAs are involved in different biological functions and pathological processes, through the special pattern of miRNA sponges, alternative splicing or transcriptional regulation, and regulating gene expression.^22^, ^23^, ^24^ Studies have shown that there are a large number of abnormally expressed cricRNAs in hemangiomas,^19^, ^25^ suggesting that cricRNAs may play an important regulatory role in the tumorigenesis of hemangiomas. In our research, circAP2A2 was remarkably upregulated by microarrays and HAs tissues. In addition, in order to further verify the biological function of circAP2A2 in hemangiogenesis and development, we knocked down the expression of circAP2A2 in HemECs and HUVEC cells, and subsequent experiments in vitro showed that the downregulation of circAP2A2 expression can inhibit proliferation and cell migration of HemECs and HUVEC cells. All these finding implied that circAP2A2 might act as an oncogene in infantile hemangiomas progression.

At present, a large number of studies have shown that circRNAs can be used as competitive endogenous RNA, which are contributed to bind to miRNAs so as to regulate tumorigenesis.^11^, ^26^, ^27^, ^28^ Hansen, et al^29^ found that ciRS‐7 contains more than 70 miR‐7 binding sites and binds tightly to Argonaute protein. In another example, Cheng et al^30^ found that circTP63 competitively binds to miR‐873‐3p to abolish the inhibitory effect of miR‐873‐3p on FOXM1 and then promote cell proliferation. MiR‐382‐5p is involved in the carcinogenesis in different cancers, for example, colorectal cancer,^31^ breast cancer,^32^ and prostate cancer,^33^ and is related to the chemoresistance of cancer cells.^34^ Furthermore, miR‐1307 contributes to prostate cancer proliferation by targeting FOXO3A.^35^ In our study, miR‐382‐5p expression was confirmed to be regulated in HUVEC cells. Moreover, we confirmed the presence of a binding site for circAP2A2 and miR‐382‐5p by luciferase assay, suggesting that circAP2A2 can act as a miR‐382‐5p sponge to regulate downstream mRNA of miR‐382‐5p.

VEGFA levels are significantly elevated in the proliferative phase of infantile hemangiomas, and gradually decrease in the regression phase, which is highly correlated with the development of infantile hemangiomas.^36^ Also, it is well known that the VEGFA‐mediated VEGFA/VEGFR pathway is an important signaling pathway involved in the development of hemangiomas, and also VEGFA interacts with HIF‐1α to regulate angiogenesis.^37^ In our research, bioinformatics analysis revealed that VEGFA is a miR‐382‐5p target. Also, it has been confirmed that the inhibition of circAP2A2 could downregulate VEGFA expression in HemECs and HUVEC cells in vitro. In the meanwhile, the changed expression of VEGFA caused by circAP2A2 could be reversed by miR‐382‐5p inhibitor, which is in accordance with the conversion of enhanced cell proliferation in HemECs and HUVEC cell lines. Therefore, our studies indicated that circAP2A2 can regulate the occurrence of hemangiomas through the circAP2A2/miR‐382‐5p/VEGFA axis.

In conclusion, our study confirms upregulation of circAP2A2 in HC, and also proposes a key role of circAP2A2 in IH, functioned as ceRNA by regulating the expression of VEGFA via sponge miR‐382‐5p and participating in IH progression. The study may provide a new potential target for IH diagnosis and treatment.
